# Application of NanoString Technologies in Chronic Myeloid Leukemia, Essential Thrombocythemia, Primary Myelofibrosis, and Polycythemia Vera: A Pilot Study

**DOI:** 10.3390/diagnostics16111725

**Published:** 2026-06-03

**Authors:** Jun-Hyung Bae, Kyung-Jin Bae, Chi-Hyun Cho

**Affiliations:** 1 Department of Medicine, Korea University College of Medicine, 73, Goryeodae-ro, Seongbuk-gu, Seoul 02841, Republic of Korea; reecebaejun@naver.com (J.-H.B.); qorudwls10@korea.ac.kr (K.-J.B.); 2 Department of Laboratory Medicine, College of Medicine, Korea University Ansan Hospital, 123 Jeokgeum-ro, Danwon-gu, Ansan-si 15355, Gyeonggi-do, Republic of Korea

**Keywords:** myeloproliferative neoplasms, NanoString, cytokine signaling, bone marrow mononuclear cells, differential gene expression, immune pathways

## Abstract

**Background/Objectives**: Chronic myeloid leukemia (CML), essential thrombocythemia (ET), primary myelofibrosis (PMF), and polycythemia vera (PV) are myeloproliferative neoplasms (MPNs) that require precise molecular characterization. Although driver mutations such as BCR-ABL1 and JAK2 are diagnostically important, they do not fully explain disease heterogeneity. The NanoString nCounter^®^ system enables direct multiplex gene expression analysis without RNA amplification and is suitable for degraded bone marrow specimens. This study aimed to analyze cytokine gene expression in bone marrow mononuclear cells of patients with MPNs and controls using NanoString technology, identify differentially expressed genes (DEGs) among MPN subtypes, and investigate their biological significance. **Methods**: Bone marrow aspirates were collected from 19 patients with MPNs (CML, ET, PMF, and PV) and 6 control patients. Mononuclear cells were isolated, and RNA expression of a 40-gene cytokine panel was analyzed using the NanoString nCounter^®^ system with strict quality control and normalization. DEGs were identified for each MPN subtype, followed by Gene Ontology(GO) and Kyoto Encyclopedia of Genes and Genomes(KEGG) pathway analyses. **Results**: CML and PV demonstrated 20 and 12 DEGs, respectively. In contrast, ET showed only one DEG (IRAK2), and PMF showed none. Functional analyses revealed enrichment of cytokine signaling, Toll-like receptor (TLR), and JAK-STAT pathways in CML, indicating immune and inflammatory dysregulation. PV DEGs were associated with TLR signaling, IL-17 pathways, and cytokine–cytokine receptor interactions, suggesting active cytokine-mediated inflammation. **Conclusions:** CML and PV exhibited distinct cytokine-driven transcriptional signatures, whereas ET and PMF exhibited minimal alterations. These findings support the clinical utility of NanoString technology for bone marrow specimens and highlight disease-specific immune pathways as potential diagnostic biomarkers in MPNs.

## 1. Introduction

Chronic myeloid leukemia (CML), essential thrombocythemia (ET), primary myelofibrosis (PMF), and polycythemia vera (PV) are classified as myeloproliferative neoplasms (MPNs), a group of hematological malignancies characterized by clonal proliferation of myeloid lineage cells in the bone marrow (BM) [1]. Accurate diagnosis and subclassification of these disorders are essential for appropriate treatment and prognostic prediction and rely heavily on the integration of clinical, morphologic, cytogenetic, and molecular data. Common molecular alterations such as BCR-ABL1 fusion in CML and JAK2, CALR, or MPL mutations in BCR-ABL1-negative MPNs serve as key diagnostic markers [2,3]. However, these mutations do not fully explain disease heterogeneity or prognosis, particularly for ET and PMF [3].

Recently, transcriptome-based profiling has emerged as a powerful tool for investigating disease mechanisms and identifying potential biomarkers. However, the application of gene expression assays to liquid specimens, such as peripheral blood or BM aspirates, remains technically challenging because of RNA degradation, variable quality, and the presence of mixed cell populations. Conventional methods, such as RNA sequencing, often require high-quality RNA and may not be feasible in routine clinical practice.

The NanoString nCounter^®^ platform provides a promising alternative for transcriptomic analysis of hematological malignancies. This digital barcode-based system enables direct quantification of RNA molecules without amplification, making it particularly suitable for partially degraded RNA obtained from BM aspirates [4]. Moreover, NanoString technology has been validated for use in clinical settings owing to its robustness, reproducibility, and multiplexing capabilities [5,6].

In this study, we investigated the utility of NanoString technology for cytokine gene expression profiling in BM mononuclear cells (MNCs) from patients with CML, ET, PMF, and PV. Residual BM aspirates from patients with MPNs and control patients were collected and processed for RNA extraction and subsequent hybridization using a 40-gene NanoString cytokine panel. This study aimed to identify differentially expressed genes (DEGs) across MPN subtypes and evaluate their biological relevance through functional annotation and pathway enrichment analyses. Our goal is to uncover the novel molecular features of MPNs and assess the clinical utility of NanoString technology in the molecular analysis of BM aspirate specimens.

## 2. Materials and Methods

### 2.1. Sample Collection and Preparation

Between May 2018 and June 2019, 25 patients were enrolled in the study after providing informed consent. Residual aliquots of BM aspirates were obtained from individuals undergoing BM examinations for the diagnosis of hematological malignancies. Based on BM smear findings, pathological review, and application of the World Health Organization diagnostic criteria, patients were categorized into CML, ET, PMF, or PV groups. The control cohort (*n* = 6) comprised patients with lymphoma without BM involvement [7]. None of the participants presented with active infections, inflammatory disorders, or renal failure. The study protocol was approved by the Institutional Review Board (IRB) of Korea University Ansan Hospital (IRB number: 2018AS0256) and conducted in accordance with the principles of the Declaration of Helsinki.

BM aspirates were collected in ethylenediaminetetraacetic acid-containing BD Vacutainer tubes (Becton Dickinson, Franklin, NJ, USA) and centrifuged at 2399× *g* for 10 min. The BM aspirate infranatant fraction containing hematopoietic cells was subsequently used for MNC isolation.

### 2.2. MNC Isolation

MNCs were isolated from BM aspirates using Lymphoprep in SepMate tubes (STEMCELL Technologies, Vancouver, BC, Canada) [8]. Briefly, 4.5 mL of Lymphoprep (density: 1.077 g/mL; STEMCELL Technologies) was added to 15 mL SepMate tubes. BM aspirates were diluted 1:1 with phosphate-buffered saline (PBS) and gently mixed before being layered along the sides of vertically positioned tubes. Samples were centrifuged at 1200× *g* for 10 min, and the upper MNC layer was transferred to a new 15 mL conical tube (Becton Dickinson), washed with PBS to a final volume of 14.5 mL, and centrifuged at 300× *g* for 8 min. The pellet was resuspended in 2 mL PBS, aliquoted into 1.5 mL tubes, and centrifuged at 400× *g* for 5 min. The final BM MNC pellet was stored at −80 °C until mRNA extraction.

### 2.3. mRNA Extraction from BM MNCs

The frozen cell pellets were thawed gradually in a pre-cooled bead bath. Subsequently, 1 mL of Trizol reagent (#79306-200 mL; Qiagen, Valencia, CA, USA) was added to the thawed samples, followed by brief vortexing and incubation for 5 min at room temperature (20–24 °C). Chloroform (200 μL; #34854-1 L; Sigma Aldrich, St. Louis, MO, USA) was then added, and the mixture was shaken for 15 s and incubated for another 5 min at 20–24 °C. Following centrifugation at 13,000× *g* for 15 min at 4 °C, the aqueous phase was carefully transferred to fresh 1.5 mL tubes, to which an equal volume of isopropanol was added. The samples were gently inverted four times, incubated for 10 min at 20–24 °C, and centrifuged again at 13,000× *g* for 10 min at 4 °C. The resulting supernatant was removed, and the RNA pellets were washed twice with 1 mL of 70% ethanol, each followed by centrifugation at 7500× *g* for 5 min at 4 °C. After air-drying, RNA pellets were dissolved in 10–20 μL of RNase-free water and transferred to new 1.5 mL tubes, and RNA integrity was assessed using the 2100 Bioanalyzer capillary electrophoresis system (Agilent Technologies, Santa Clara, CA, USA).

### 2.4. mRNA Expression Analysis

The RNA samples were analyzed using the nCounter Analysis System (NanoString Technologies Inc., Seattle, WA, USA) in accordance with the manufacturer’s protocol. Cytokine gene panels were used, including the following targets: CXCL1, CXCL2, CXCL10, IL1A, IL1B, IL2, IL3, IL4, IL5, IL6, IL7, IL8, IL9, IL10, IL11, IL18, IL28A/B, IL29, IL1R1, IL1R2, IRAK1, IRAK2, IRAK4, TRAF3, TRAF6, NF-κB, STAT1, STAT2, STAT3, STAT4, IRF7, TLR1, TLR2, TLR4, TLR5, CASP8, FOS, ELK1, CRP, and NGAL. These specific 40 genes were logically selected as a focused subset from a comprehensive commercial human inflammation panel. Given that hundreds of cytokines exist, this subset was strategically curated to evaluate the core components of BM microenvironmental inflammation, focusing on key pro-inflammatory/anti-inflammatory cytokines and chemokines, along with their upstream receptors and downstream signaling nodes (specifically the TLR/NF-κB and JAK–STAT pathways) that are deeply implicated in the pathobiology, clonal proliferation, and immune evasion of MPN.

For hybridization, a reaction mixture was assembled by combining 5 μL of RNA (100–300 ng), 8 μL of Master Mix (reporter CodeSet and hybridization buffer), and 2 μL of capture probeSet. The mixture was briefly spun down and incubated at 65 °C in a thermocycler (Bio-Rad Laboratories Inc., Hercules, CA, USA) for 16 h (up to 48 h). Post-hybridization processing was carried out using the nCounter Prep Station (NanoString Technologies Inc.) with the appropriate Master Kit and cartridge. Each run processed 12 lanes, requiring approximately 2.5–3 h. The cartridges were analyzed using a Digital Analyzer (NanoString Technologies Inc.) with imaging across 555 fields of view.

Raw data quality was assessed according to predefined QC metrics: (i) imaging QC with a field of view > 75%, (ii) binding density QC between 0.1 and 2.25, (iii) positive control linearity with R^2^ > 0.95, and (iv) limit of detection QC where the 0.5 fM positive control probe exceeded the mean of the negative controls by more than two standard deviations. Data normalization was conducted using CodeSet control probes, and the normalized expression levels of 40 genes were used for statistical analysis.

### 2.5. Data Normalization and Statistical Analysis

Gene expression was normalized to housekeeping genes using the geNorm algorithm [9] implemented in nCounter Advanced Analysis version 2.0.134 (NanoString Technologies, USA). DEGs between two biological conditions (control vs. CML, control vs. ET, control vs. PMF, and control vs. PV) were identified using default parameters. Genes showing significant changes in expression (|Fold Change| > 2 and *p*-value < 0.05) were clustered with nCounter Advanced Analysis version 2.0.134. The DEG statistical plots were generated in R (version 4.3.0, R Foundation for Statistical Computing, Vienna, Austria) using the ggplot2 package [9]. Heatmap clustering of DEGs was performed on log_2_-transformed normalized counts using the gplots package (v3.2.0; https://cran.r-project.org/web/packages/gplots/index.html, accessed on 20 January 2026) with average-linkage hierarchical clustering.

Artificial intelligence tools, including ChatGPT based on GPT-5 (OpenAI, San Francisco, CA, USA) and Gemini (Google LLC, Mountain View, CA, USA; Gemini 3 Flash), were used solely for English language editing and the improvement of manuscript clarity and structure. No AI tools were utilized for data generation, statistical analysis, or the independent interpretation of the research results.

### 2.6. Gene Ontology Enrichment and Pathway Analysis

To explore the biological roles of DEGs identified between the control and disease groups, functional enrichment analyses were carried out, including Gene Ontology (GO) categories—biological process (BP), cellular component (CC), and molecular function (MF)—as well as Kyoto Encyclopedia of Genes and Genomes (KEGG) pathway analysis. These analyses were performed using Metascape (http://metascape.org/gp/index.html, accessed on 20 January 2026) with the default settings (minimum gene overlap ≥ 3, enrichment factor ≥ 1.5, and *p*-value < 0.01). The KEGG database was established to characterize functional interactions of genes and proteins within biological pathways [10]. The lists of upregulated and downregulated genes identified from DEG analysis were used as inputs. Enrichment analyses were performed using the KEGG_2021_human and GO_Biological_Process_2021 libraries.

## 3. Results

### 3.1. RNA Quality and the Characteristics of 40 Genes

As shown in Table 1, the total RNA extracted from BM MNCs was evaluated for quality using the BioAnalyzer System. The RNA integrity numbers (RINs) ranged from 2.0 to 6.8, indicating that the RNA purified from the BM MNCs was highly degraded. Fragmented RNA is generally suitable for application in the NanoString system, which utilizes probe hybridization.

The 40 genes used in this study can be classified according to their functions as cytokines and chemokines, cytokine/inflammatory signaling regulators, pathogen recognition receptors (innate immunity), regulators of cell death and survival, and markers of inflammatory/immune responses (Table 2).

### 3.2. DEG Profiling of CML, ET, PMF, and PV Using NanoString System Output

NanoString nCounter advanced analysis of the CML and control groups revealed that 14 genes were upregulated and six genes were downregulated in the CML group (Table 3).

**Figure 1:**
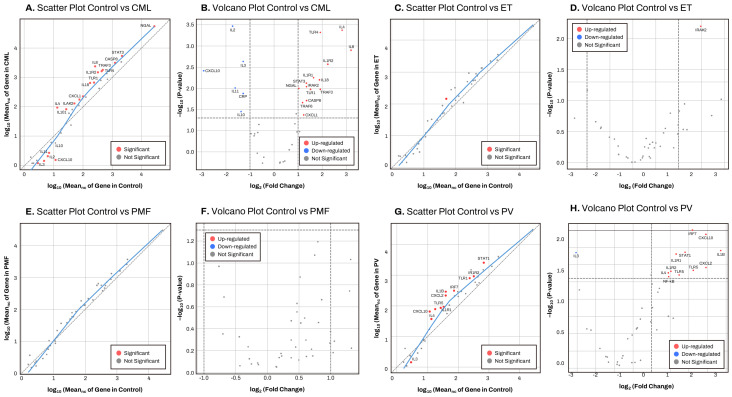
Digital multiplexed gene expression analysis for CML, ET, PMF, and PV using NanoString technology. The results of statistical data analysis were visualized via scatter and volcano plots. Red dots in the scatter plots (**A**,**C**,**E**,**G**) and volcano plots (**B**,**D**,**F**,**H**) represent significantly up- or downregulated genes in CML, ET, PMF, and PV (*p*-value < 0.05 and log_2_|FC| ≥ 1), respectively. In the scatter plots, the blue solid line indicates the regression line, while the gray dashed diagonal line represents the line of equality (y = x). In the volcano plots, the vertical dashed lines indicate the fold-change thresholds (log_2_|FC| = ±1), and the horizontal dashed line indicates the statistical significance threshold (*p* = 0.05). Abbreviations: CML, chronic myeloid leukemia; ET, essential thrombocythemia; MNC, mononuclear cell; PMF, primary myelofibrosis; PV, polycythemia vera. Mean_NC_: mean normalized counts of the same gene in the control and disease groups. Fold change: fold change in normalized counts of the same gene between the control and disease groups.

**Figure 2:**
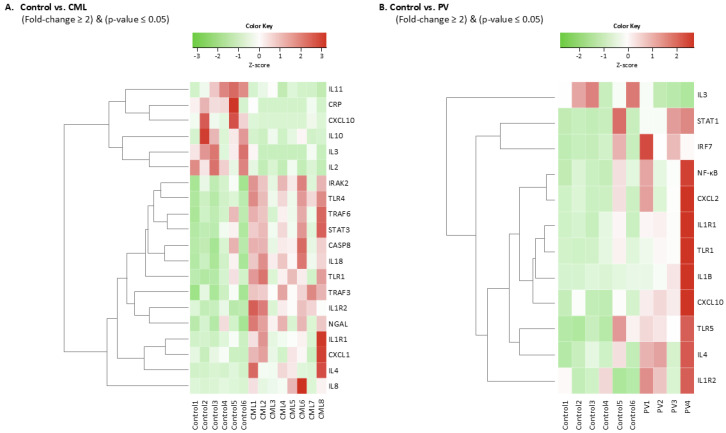
Heatmap of DEGs derived from comparison of control and myeloproliferative neoplasm (CML and PV) groups. (**A**) For CML, the heatmap of hierarchical clustering indicates differentially up (14) and downregulated (6) genes (rows) compared with the control group. (**B**) For PV, the heatmap of hierarchical clustering indicates differentially up (11) and downregulated (1) genes (rows) compared with the control group. The Intensity of the color indicates gene expression levels that were normalized. Red and green colors indicate up and downregulation, respectively. Abbreviations: DEG, differentially expressed gene; CML, chronic myeloid leukemia; PV, polycythemia vera.

The comparative results are presented as scatter and volcano plots (Figure 1A,B). Based on the Z-score of the expression values of the 20 DEGs, a heatmap was generated, and as shown in Figure 2A, a clear difference in gene expression was observed between the CML and control groups. Hierarchical clustering analysis further demonstrated that the 14 upregulated and 6 downregulated genes were grouped completely distinctly into separate clusters.

When transcriptomic profiles from four patients with ET were compared with those of the control group, only one gene, IRAK2, was found to be significantly upregulated in ET relative to controls (*p* < 0.05 and log_2_|Fold Change| ≥ 2; Table 3). No other genes exhibited significantly differential expression between the two groups. The comparative results are presented as scatter and volcano plots (Figure 1C,D). Because only a single gene met the threshold for differential expression, a heatmap analysis was not performed.

When the NanoString data obtained from three patients with PMF were compared with data from the control group, there were no upregulated or downregulated genes in PMF compared to the control group (*p* < 0.05 and log_2_|Fold-Change (FC)| ≥ 1). The comparative results are presented as scatter and volcano plots (Figure 1E,F).

When transcriptomic profiles from four patients with PV were compared with those of the control group, a total of 11 genes were significantly upregulated, and one gene was downregulated in PV compared with controls (*p* < 0.05 and log_2_|Fold Change| ≥ 2). The DEGs were visualized using scatter and volcano plots (Figure 1G,H). In addition, hierarchical clustering of these 12 DEGs showed that single downregulated genes clustered separately from the remaining 11 upregulated genes, as shown in the heatmap (Figure 2B). These findings indicate that PV is characterized by broad transcriptional activation of immune- and inflammation-related genes compared to the control group (Figure 2B, Table 2).

### 3.3. GO and KEGG Pathway Analysis of CML and PV

#### 3.3.1. GO and KEGG Pathway Analysis of CML

**Figure 3:**
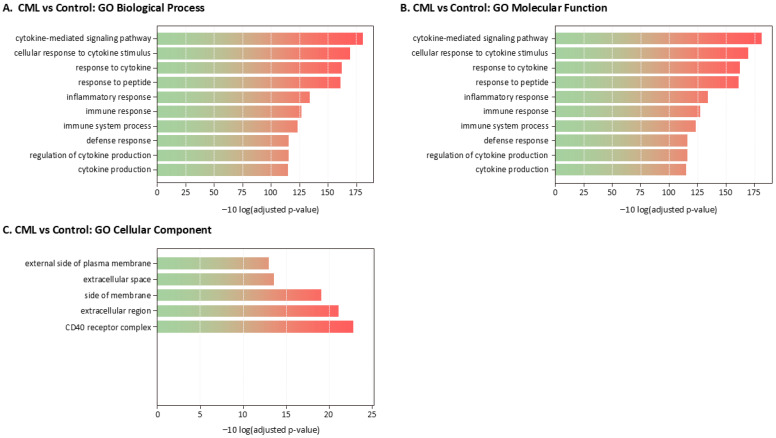
Multiple GO functional annotation enrichment analyses of DEGs in CML with the Metascape web-based bioinformatics tool. The bar plots show the top 10 significantly enriched terms in each GO category: (**A**) Biological Process, (**B**) Cellular Component, and (**C**) Molecular Function. The X-axis indicates the significance level (scored as −log10(*p*-value)), and the Y-axis lists enriched GO terms. Bars with higher values represent more significant enrichment. Abbreviations: DEG, differentially expressed gene; CML, chronic myeloid leukemia; GO, Gene Ontology.

GO analysis of the 20 DEGs was conducted using NetworkAnalyst to characterize their functional roles. This analysis highlighted the most significantly enriched biological processes, molecular functions, and cellular components associated with these genes. Biological process analysis revealed enrichment in 10 categories: cytokine-mediated signaling pathway, cellular response to cytokine stimulus, response to cytokine, response to peptide, inflammatory response, immune response, immune system process, defense response, regulation of cytokine production, and cytokine production (Figure 3A). Molecular function analysis indicated a strong association between cytokine activity and cytokine receptor binding (Figure 3B). Cellular component analysis showed that the DEGs were mainly associated with the CD40 receptor complex, extracellular space, and external side of the plasma membrane (Figure 3C).

**Figure 4:**
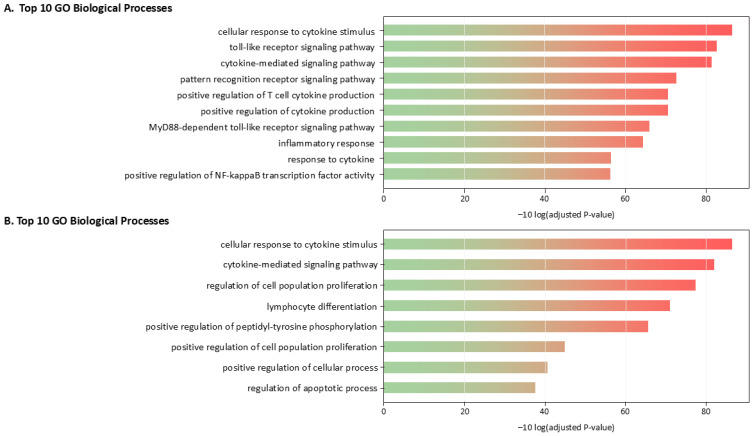
GO enrichment analysis of up and downregulated genes in CML with the MetaScape web-based bioinformatics tool. The X-axis in each graph represents the significance level (scored as −10 × log(adjusted *p*-value)). The Y-axis denotes the related GO biological process terms. Fourteen up (**A**) and six downregulated genes (**B**) were mapped at the GO biological process classification. Abbreviations: CML, chronic myeloid leukemia; GO, Gene Ontology.

GO enrichment analysis of biological processes was conducted for the 14 upregulated and six downregulated genes among the 20 DEGs. The upregulated genes were primarily associated with cytokine responses, positive regulation of cytokine production, and cytokine-mediated signaling pathways (Figure 4A). The downregulated genes were related to cytokine response, cytokine-mediated signaling, regulation of cell population proliferation, and lymphocyte differentiation (Figure 4B).

**Figure 5:**
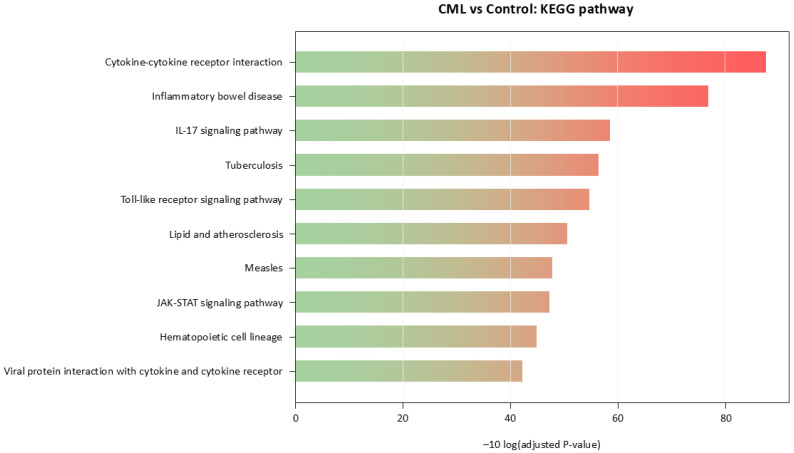
KEGG pathway analysis of DEGs in CML with the Metascape web-based bioinformatics tool. The X-axis in each graph represents the significance level (scored as −log10(adjusted *p*-value)). The Y-axis denotes the related KEGG pathways. Abbreviations: KEGG, Kyoto Encyclopedia of Genes and Genomes; DEG, differentially expressed gene; CML, chronic myeloid leukemia.

To gain further insight into the functional implications of the 20 DEGs identified in CML, we conducted a KEGG pathway enrichment analysis. This analysis revealed that CML-associated DEGs were significantly enriched in the following pathways: cytokine–cytokine receptor interaction, inflammatory bowel disease, the IL-17 signaling pathway, tuberculosis, the Toll-like receptor (TLR) signaling pathway, lipids and atherosclerosis pathway, measles, the JAK-STAT signaling pathway, hematopoietic cell lineage, and viral protein interaction with cytokines and cytokine receptors (Figure 5).

**Figure 6:**
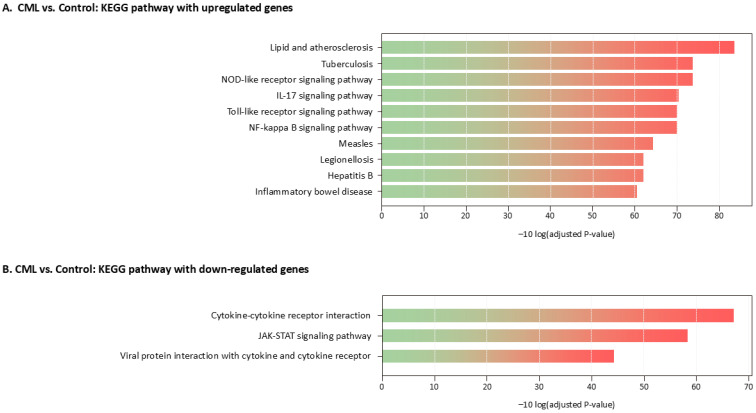
KEGG pathway analysis of up- and downregulated genes in CML with the MetaScape web-based bioinformatics tool. The X-axis in each graph represents the significance level (scored as −10 × log(adjusted *p*-value)). The Y-axis denotes the related KEGG pathway terms. Fourteen up (**A**) and six downregulated genes (**B**) were mapped at the KEGG pathway classification. Abbreviations: KEGG, Kyoto Encyclopedia of Genes and Genomes; CML, chronic myeloid leukemia.

Additionally, KEGG pathway network analysis was performed on each of the 14 upregulated and six downregulated DEGs. As shown in Figure 6A, these upregulated DEGs were associated with several immune cell signaling and disease-related pathways, including the IL-17 signaling pathway, TLR signaling pathway, lipids and atherosclerosis, tuberculosis, and measles. Six downregulated DEGs were involved in KEGG pathways such as cytokine–cytokine receptor interactions, the JAK-STAT signaling pathway, and viral protein interaction with cytokines and cytokine receptors (Figure 6B).

#### 3.3.2. GO and KEGG Pathway Analysis of PV

**Figure 7:**
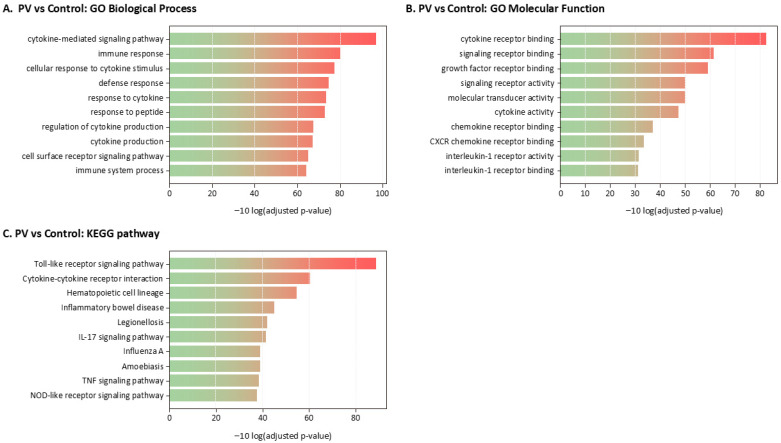
Multiple GO functional annotation enrichment and KEGG pathway analysis analyses of DEGs in PV with the Metascape web-based bioinformatics tool. The bar plots show the top 10 significantly enriched terms in each category: (**A**) GO Biological Process, (**B**) GO Molecular Function, and (**C**) KEGG pathway analysis. The X-axis indicates the significance level (scored as −10 log (adjusted *p*-value)), and the Y-axis lists enriched terms. Bars with higher values represent more significant enrichment. Abbreviations: GO, Gene Ontology; KEGG, Kyoto Encyclopedia of Genes and Genomes; PV, polycythemia vera.

GO analysis of the 12 DEGs identified in PV was conducted using Metascape and NetworkAnalyst platforms to characterize their functional significance. The analysis revealed that the most significantly enriched GO biological processes were predominantly related to immune and cytokine signaling. Specifically, 10 GO biological process categories exhibited significant enrichment, ranked by adjusted *p*-values, as follows: cytokine-mediated signaling pathway, immune response, cellular response to cytokine stimulus, defense response, response to cytokines, response to peptides, regulation of cytokine production, cytokine production, the cell surface receptor signaling pathway, and immune system processes (Figure 7A).

GO molecular function analysis indicated that the DEGs were primarily involved in cytokine and receptor interactions. The top enriched terms included cytokine receptor binding, signaling receptor binding, growth factor receptor binding, molecular transducer activity, signaling receptor activity, cytokine activity, chemokine receptor binding, CXCR chemokine receptor binding, interleukin-1 receptor activity, and interleukin-1 receptor binding (Figure 7B). These results suggest that PV is characterized by a marked activation of receptor-mediated cytokine and chemokine signaling.

To further elucidate the biological implications of these DEGs, a KEGG pathway enrichment analysis was performed. PV-associated DEGs were significantly enriched in immune- and inflammation-related pathways, including the Toll-like receptor signaling pathway, cytokine–cytokine receptor interaction, hematopoietic cell lineage, inflammatory bowel disease, Legionellosis, the IL-17 signaling pathway, Influenza A, amoebiasis, the TNF signaling pathway, and NOD-like receptor signaling pathway (Figure 7C). In particular, pathways such as TLR signaling and cytokine–cytokine receptor interactions exhibited the highest enrichment scores, indicating the activation of innate immune and inflammatory cascades.

Taken together, GO and KEGG analyses demonstrated that PV is characterized by the transcriptional upregulation of genes involved in cytokine and receptor-mediated signaling, innate immune activation, and hematopoietic regulation.

## 4. Discussion

In the CML patient cohort, 20 genes showed significant changes in expression, with 14 upregulated and 6 downregulated genes (Table 3). Among the upregulated genes, elevated TLR4, TLR1, IRAK2, TRAF6, IL1R1, and IL-18 suggest that CML promotes a chronic inflammatory state through TLR- and IL-1-receptor-mediated activation of NF-κB and JNK pathways, supporting autonomous survival signaling and modulation of the microenvironment [11,12].

Increased STAT3 and TRAF3 expression further reflects dysregulated BCR-ABL-associated signaling, contributing to abnormal proliferation and resistance to apoptosis [13]. Upregulation of CXCL1 and IL-8 implies enhanced recruitment of inflammatory cells and the activation of pathways involved in tumor survival and angiogenesis [14].

The downregulated genes suggested weakened immune surveillance and a shift toward a more permissive immune microenvironment (Table 3). Reduced expression of IL-2 and CXCL10 indicates diminished cytotoxic immune activity, including that of T and NK cells, implying an overall decline in immune surveillance capacity [15,16].

In contrast to CML, the expression profile of BM MNCs in ET was characterized by a remarkable paucity of DEG (Table 3). Only IRAK2 was significantly upregulated compared with controls (Table 3). IRAK2 encodes a kinase essential for the IL-1 receptor (IL-1R)/TLR signaling cascade, mediating MyD88-dependent activation of downstream inflammatory effectors such as NF-κB [17]. This isolated upregulation suggests that while ET may not exhibit the broad inflammatory activation seen in CML, subtle priming of innate immune signaling nodes may exist [18]. This finding aligns with the concept that ET is relatively ‘cytokine-silent’ at the transcriptional level in BM, although prior studies on plasma cytokines have reported heterogeneous inflammatory signatures influenced by JAK2V617F status and clinical history of thrombosis [19,20]. Consequently, our data imply that inflammatory dysregulation in ET may be less driven by global transcriptional reprogramming of MNCs and more dependent on specific context-dependent signaling or other cell fractions that were not captured in this analysis.

Conversely, PV exhibited a robust inflammatory transcriptomic signature characterized by the broad activation of immune- and inflammation-related genes. Relative to the controls, 11 genes were upregulated, and one gene was downregulated (Table 3; Figure 1G,H and Figure 2B). The upregulated gene set revealed the concurrent activation of multiple inflammatory axes: (i) the IL-1 signaling module, evidenced by the upregulation of IL1β and its receptors (IL1R1, IL1R2) [21]; (ii) innate immune sensing via TLR1 and TLR5 [22,23]; and (iii) interferon- and chemokine-mediated responses, reflected by increased expression of STAT1 [24], IRF7 [25], CXCL10 [26], and CXCL2 [27]. Notably, IL3 was the only downregulated gene that formed a distinct cluster separate from that of the inflammatory module (Table 3, Figure 2B).

Collectively, these results indicate that the BM in PV creates a highly inflammatory microenvironment driven by cytokine receptor circuits and innate immune activation. This is consistent with clinical observations correlating abnormal cytokine milieus in PV with constitutional symptoms and thrombotic risk [28]. Furthermore, mechanistic studies in JAK2V617F-driven murine models have identified IL-1β as a critical mediator of myelofibrotic transformation, suggesting that the IL1β/IL1R upregulation observed in our cohort may represent a biologically significant driver of disease progression rather than a mere bystander effect [29].

To further interpret the functional implications of these subtype-specific DEGs, we performed GO and KEGG pathway analyses, focusing on the biological processes and signaling networks associated with the dysregulated gene sets.

For CML, in the GO analysis of biological processes for upregulated genes (Figure 4A), the top 10 enriched terms were predominantly associated with inflammation and immune signaling. This indicated that CML cells either continuously secreted inflammatory cytokines or were in a state of heightened sensitivity to external stimuli.

The terms “*cellular response to cytokine stimulus*” and “*cytokine-mediated signaling pathway*” suggest that the cells actively respond to inflammatory cues, potentially contributing to the establishment of a tumor microenvironment favorable for cancer cell survival.

Inflammatory cytokines can activate signaling pathways such as STAT3 and NF-κB within cancer cells, thereby promoting proliferation and suppressing apoptosis. In particular, the term “*positive regulation of NF-κB transcription factor activity*” indicates activation of NF-κB, a key regulator known to enhance cell survival and inhibit programmed cell death, underscoring a signaling axis that strongly supports cancer cell persistence [30].

Enrichment of innate immune-related pathways such as the “*Toll-like receptor signaling pathway*,” “*pattern recognition receptor signaling pathway*,” and “*MyD88-dependent Toll-like receptor signaling pathway*” further indicates aberrant activation of inflammatory signaling in CML (Figure 4A). TLRs and their downstream adaptor protein MyD88 recognize pathogen-associated molecular patterns (PAMPs) or damage-associated molecular patterns (DAMPs), inducing the secretion of inflammatory cytokines and chemokines [31]. Such sustained inflammatory signals may create autocrine and paracrine conditions that favor the survival and expansion of leukemic cells.

CML is characterized by the presence of the BCR-ABL1 oncogene, which encodes a constitutively active fusion protein with aberrant tyrosine kinase activity. This fusion protein activates multiple downstream signaling pathways, including RAS and JAK/STAT, which promote cellular proliferation, inhibit differentiation, and prevent apoptosis [13]. Among these, the activation of the transcription factor NF-κB leads to the expression of pro-inflammatory genes, which may contribute to the upregulation of TLR signaling pathways [32,33].

In the GO analysis of biological processes for the downregulated genes (Figure 4B), the results reflected a loss of regulatory control and enhanced survival capacity, which are hallmark features of cancer. The downregulation of “*regulation of apoptotic process*” and “*regulation of cell population proliferation*” indicates disruption of the mechanisms that normally restrain proliferation and promote programmed cell death, supporting the uncontrolled, continuous growth driven by oncogenes such as BCR-ABL.

The reduced expression of “*lymphocyte differentiation*” suggests impaired differentiation of lymphoid lineages, including T and B cells (Figure 4B). This implies that the development of adaptive immune cells capable of targeting CML cells is hindered, whereas CML cells continue to exploit the inflammatory responses to their advantage.

Notably, “*cellular response to cytokine stimulus*” and “*cytokine-mediated signaling pathway*” appear among not only the upregulated (Figure 4A) but also the downregulated categories (Figure 4B). This indicates that cytokine signaling in CML is not uniformly activated or suppressed; rather, it is selectively modulated, amplifying signals favorable to CML cell survival while attenuating those that are detrimental, reflecting a dysregulated and highly biased cytokine signaling landscape.

KEGG pathway analysis of the DEGs in CML showed that they were overrepresented in pathways related to intercellular immune signaling and inflammatory responses (Figure 5). KEGG pathway analysis of the upregulated genes (Figure 6A) was consistent with the GO biological process analysis (Figure 4A) and showed that the “*Toll-like receptor signaling pathway*” and “*NF-κB signaling pathway*” were prominently enriched, indicating strong activation of innate immune and inflammatory signaling. Pathways such as the “*IL-17 signaling pathway*” further suggest the presence of an inflammatory cytokine milieu that indirectly supports cancer cell survival and proliferation.

The enrichment of pathways labeled “*Tuberculosis*,” “*Measles*,” “*Legionellosis*,” and “*Hepatitis B*” (Figure 6A) is likely attributable to the upregulation of innate immune and inflammation-related genes (e.g., NF-κB, TLRs, and NOD family genes) that are shared across these pathogen-associated pathways, rather than implying direct relevance to infection [10,13].

In addition, genes involved in “*Lipid and atherosclerosis*” (Figure 6A) were significantly enriched, suggesting aberrant metabolic remodeling in CML. Such alterations may provide the energy substrates and membrane components needed to sustain the rapid proliferation and survival of leukemic cells.

As for CML, in the KEGG pathway analysis of downregulated genes (Figure 6B), “*Cytokine–cytokine receptor interaction*” may suggest not a global suppression of all receptor-mediated signaling, but rather a selective inhibition of specific cytokine cues and a reduction in external signal dependency. The enrichment of “*Viral protein interaction with cytokine and cytokine receptor*” can also be interpreted as a secondary consequence of the selective repression of the cytokine pathway.

A notable finding was the downregulation of multiple genes associated with the “*JAK–STAT signaling pathway*”. This result may be related to the finding that CML cells suppress the normal cytokine-dependent JAK–STAT signaling axis while shifting toward a BCR-ABL1-driven, aberrant STAT activation program [13,34]. This signaling rewiring allows leukemic cells to retain only the survival-promoting outputs of STAT activity, while silencing regulatory pathways associated with normal cytokine responsiveness, thereby contributing to the malignant acquisition of autonomous proliferative capacity.

Regarding PV, corroborating the inflammatory DEG profile, GO enrichment analysis demonstrated that PV-associated transcripts were predominantly mapped to immune response and inflammatory pathways, implying that dysregulated cytokine signaling is a key molecular feature of PV (Figure 7A,B). Enriched biological process terms including “*cytokine-mediated signaling pathway*,” “*immune response*,” and “*regulation of cytokine production*” underscore a transcriptional state poised for heightened cytokine responsiveness and autocrine/paracrine amplification (Figure 7A). In parallel, molecular function analysis highlighted “*cytokine receptor binding*” and “*interleukin-1 receptor activity*,” directly reflecting the upregulation of the IL1B ligand and its cognate receptors (IL1R1, IL1R2) (Figure 7B). These data suggest that receptor–ligand engagement, particularly within the IL-1 axis, constitutes a central component of the PV pathobiology.

KEGG pathway analysis further reinforced the dominance of innate immune networks in PV (Figure 7C), showing enrichment for “*Toll-like receptor signaling*,” “*Cytokine–cytokine receptor interaction*,” “*TNF signaling*,” and “*IL-17 signaling.*” These pathways are mechanistically coherent with the observed gene expression changes: the TLR1/TLR5-NF-κB-IL1B/IL1R axis provides a plausible basis for the enrichment of TLR and cytokine interaction pathways, while chemokines such as CXCL2 and CXCL10 support the recruitment of immune effectors. The identification of shared inflammatory signaling modules, characterized by enriched TLR signaling, cytokine–cytokine receptor interactions, the IL-1 signaling axis, and increased expression of inflammatory chemokines such as CXCL2 and CXCL10, reflects pathway crosstalk converging on NF-κB activation and cytokine amplification loops, thereby sustaining the chronic inflammatory microenvironment characteristic of PV [19,22,29].

Taken together, the combined DEG and pathway analyses indicate that PV is characterized not by isolated cytokine fluctuations, but by a coordinated activation of innate immune sensing and cytokine–receptor signaling. This supports a model in which chronic inflammatory signaling and immune-hematopoietic crosstalk are integral to the maintenance and clinical phenotype of PV.

Despite the meaningful insights into the transcriptomic landscapes of MPNs, this study has a clear limitation regarding its small cohort size (CML, n = 8; PV, n = 4; ET, n = 4; and PMF, n = 3). Due to the extremely limited sample size, the statistical power was likely insufficient to capture the full spectrum of transcriptional changes, which potentially explains the remarkable paucity of DEGs observed in ET (n = 1) and the absence of DEGs in PMF (n = 0). Therefore, these findings must be interpreted with caution as a pilot and exploratory analysis in nature, rather than as definitive biological characteristics of the broader MPN population. Future large-scale studies with larger, independent cohorts are warranted to validate these subtype-specific inflammatory signatures and generalize the conclusions to clinical settings.

Another technological limitation is the lack of orthogonal validation using alternative methodologies, such as RT-qPCR for mRNA levels or ELISA/multiplex assays for protein expression in bone marrow supernatant or plasma. Although the NanoString nCounter platform is widely recognized for its high digital profiling accuracy and strong correlation with standard RT-qPCR without the need for enzymatic amplification [35], the lack of cross-platform validation remains a limitation of this study. Consequently, further validation studies utilizing orthogonal laboratory techniques in a larger, independent cohort will be essential to definitively confirm the clinical and biological relevance of the key subtype-specific DEGs identified in this exploratory work.

Furthermore, methodological differences between soluble cytokine measurement and cytokine mRNA detection should be considered when interpreting inflammatory profiles across different MPN subtypes. For example, cytokine mRNA profiling has shown relatively strong discriminatory performance in CML and PV. In contrast, mRNA-based analyses demonstrated reduced sensitivity in PMF, identifying fewer DEGs in our study. This discrepancy likely arises because PMF is characterized by a heavily altered bone marrow microenvironment where massive amounts of secreted cytokine proteins have already accumulated [36]. In such highly fibrotic and protein-saturated environments, cellular transcription (mRNA expression) may not reflect the actual extracellular cytokines due to potential negative feedback loops or altered cellular kinetics. Soluble cytokine analysis of bone marrow supernatant or plasma may be inherently better suited for capturing the extensive inflammatory burden characteristic of PMF [36,37].

These findings underscore the biological and technical differences between the two methodologies. While transcriptomic approaches such as NanoString-based profiling provide high reproducibility, robust analytical performance, and reduced susceptibility to matrix-related effects, discrepancies between gene expression and the actual systemic inflammatory milieu may occur due to multiple biological and pathophysiological factors [38,39]. In this regard, soluble cytokine measurements may more directly reflect the functional secretory inflammatory milieu. Accordingly, each modality possesses distinct advantages and limitations, suggesting that soluble cytokine and cytokine mRNA analyses may provide complementary insights depending on the specific MPN subtype under investigation. Furthermore, integrating these transcriptomic profiles with bone marrow flow cytometry for cellular immunophenotyping may offer a synergistic approach to mitigate overlapping signatures and enhance diagnostic reliability in future expanded cohorts.

In conclusion, we applied the NanoString nCounter advanced analysis to the CML, ET, PMF, and PV groups, and the observed results were consistent with those of previous studies in all groups except PMF, where no DEGs were detected. Notably, GO and KEGG pathway analyses in the CML group highlighted key mechanisms and potential therapeutic targets, such as the JAK-STAT and IL-17 pathways. In addition, PV was characterized by coordinated transcriptional activation of innate immune sensing and cytokine–receptor signaling—particularly involving the IL-1 axis and TLR/NF-κB pathways. Using NanoString analysis, our understanding of these diseases has advanced further, and future studies may utilize these findings to explore different classifications of hematological cancers and develop targeted and effective therapeutic strategies.

## Figures and Tables

**Table 1:** Clinical information and quality control of total RNA extracted from BM MNCs.

ID	Sex	Age, Years	Spectrophotometer			BioAnalyzer		
			RNA Conc. (ng/µL)	A260/280	A260/230	Conc. (ng/µL)	RIN	50–300 nt%
CML#1	Female	39	926.40	1.93	1.92	926.4	2.7	57.0
CML#2	Male	50	1418.00	1.95	2.18	1418.0	2.9	47.0
CML#3	Female	81	208.70	1.75	1.15	208.7	3.4	60.0
CML#4	Male	29	272.60	1.78	1.87	272.6	3.5	55.0
CML#5	Male	63	583.40	1.87	1.71	583.4	4.2	14.0
CML#6	Male	32	109.90	1.61	0.66	109.9	5.5	55.0
CML#7	Male	28	39.80	1.67	0.10	39.8	2.8	50.0
CML#8	Female	39	79.20	1.59	0.54	79.2	6.8	20.0
PV#1	Female	62	143.60	1.70	0.41	143.6	2.6	52.0
PV#2	Male	62	291.60	1.73	2.05	291.6	2.3	50.0
PV#3	Female	63	6205.20	1.10	1.04	6205.2	2.2	46.0
PV#4	Male	50	1627.60	1.98	2.21	1627.6	2.8	51.0
PMF#1	Female	72	938.10	1.89	1.85	938.1	2.6	55.0
PMF#2	Female	50	1830.70	1.95	1.86	1830.7	2.0	64.0
PMF#3	Female	62	39.20	1.63	0.19	39.2	5.6	37.0
ET#1	Male	78	1951.90	1.96	2.24	1951.9	2.0	55.0
ET#2	Male	60	1895.70	1.96	2.27	1895.7	2.2	74.0
ET#3	Female	36	1414.60	1.93	2.27	1414.6	2.4	51.0
ET#4	Female	84	328.10	1.80	2.07	328.1	3.1	44.0
Control#1	Male	66	612.80	1.86	1.88	612.8	2.5	57.0
Control#2	Female	63	410.60	1.86	1.53	410.6	2.5	47.0
Control #3	Male	47	343.90	1.80	2.12	343.9	4.3	38.0
Control #4	Male	41	1.80	1.47	0.07	1.8	N/A	38.0
Control #5	Male	72	750.40	1.88	2.11	750.4	2.8	46.0
Control #6	Male	74	547.50	1.86	1.54	547.5	2.6	55.0

Abbreviations: BM, bone marrow; CML, chronic myeloid leukemia; ET, essential thrombocythemia; MNC, mononuclear cell; PMF, primary myelofibrosis; PV, polycythemia vera; N/A, not available.

**Table 2:** Characteristics of 40 genes included in this study.

Function Group	Genes	Description
Cytokines and Chemokines (Immune Cell Signaling)	*CXCL1*, *CXCL2*, *CXCL10*	Chemokines; guide migration of immune cells
	*IL1A*, *IL1B*, *IL2*, *IL3*, *IL4*, *IL5*, *IL6*, *IL7*, *IL8*, *IL9*, *IL10*, *IL11*, *IL18*, *IL28A/B*, *IL29*	Mediate inflammatory and immune responses (interleukins)
	*IL1R1*, *IL1R2*	IL-1 signaling receptors
Cytokine/Inflammatory Signaling Regulation	*IRAK1*, *IRAK2*, *IRAK4*	Regulate intracellular signaling downstream of IL-1/TLR pathways
	*TRAF3*, *TRAF6*	TNF receptor signaling mediators, NF-κB pathway regulators
	*NF*-κ*B*	Transcription factor regulating inflammation and immune-related gene expression
	*STAT1*, *STAT2*, *STAT3*, *STAT4*	JAK-STAT pathway transcription factors; signal transduction and transcription regulation
	*IRF7*	Interferon regulatory factor; antiviral immune responses
Pathogen Recognition Receptors (Innate Immunity)	*TLR1*, *TLR2*, *TLR4*, *TLR5*	Pathogen recognition; induction of innate immune responses
Regulators of Cell Death and Survival	*CASP8*	Induces apoptosis (programmed cell death)
	*FOS*	Transcription factor; regulates cell differentiation and activation
	*ELK1*	Transcription factor downstream of MAPK pathway
Markers of Inflammatory/Immune Responses	*CRP*	Acute-phase protein; biomarker of inflammation
	*NGAL*	Protein associated with inflammatory and immune responses

Abbreviations: CASP8, Caspase 8; CRP, C-reactive protein; CXCL2, C-X-C motif chemokine ligand 2; CXCL10, C-X-C motif chemokine ligand 10; IL1B, Interleukin 1 beta; IL1R1, Interleukin 1 receptor type 1; IL1R2, Interleukin 1 receptor type 2; IL2, Interleukin 2; IRAK1, Interleukin-1-receptor-associated kinase 1; IRF7, Interferon regulatory factor 7; NGAL, Neutrophil-gelatinase-associated lipocalin; NF-κB, Nuclear factor kappa-light-chain-enhancer of activated B cells; STAT1, Signal transducer and activator of transcription 1; TLR1, Toll-like receptor 1; TRAF3, Tumor-Necrosis-Factor-receptor-associated factor 3.

**Table 3:** List of DEGs in CML, ET, and PV, showing significant changes in expression (|Fold Change| > 2 and *p*-value < 0.05).

CML	14 Up-regulated genes	IL4	TLR4	IL8	IL1R2	IL1R1	IL18
		STAT3	IRAK2	NGAL	TLR1	TRAF3	CASP8
		TRAF6	CXCL1				
	6 Down-regulated genes	IL2	IL3	IL11	CRP	IL10	CXCL10
ET	1 Up-regulated genes	IRAK2					
PV	11 Up-regulated genes	NF-κB	TLR1	IL4	IL1R2	TLR5	CXCL2
		IL1R1	STAT1	IL1B	CXCL10	IRF7	
	1 Down-regulated genes	IL3					

Abbreviations: CASP8, Caspase 8; CML, Chronic myeloid leukemia; CRP, C-reactive protein; CXCL2, C-X-C motif chemokine ligand 2; CXCL10, C-X-C motif chemokine ligand 10; DEG, Differentially expressed gene; ET, Essential thrombocythemia; IL1B, Interleukin 1 beta; IL1R1, Interleukin 1 receptor type 1; IL1R2, Interleukin 1 receptor type 2; IL2, Interleukin 2; IRAK1, Interleukin-1-receptor-associated kinase 1; IRF7, Interferon regulatory factor 7; NGAL, Neutrophil-gelatinase-associated lipocalin; NF-κB, Nuclear factor kappa-light-chain-enhancer of activated B cells; PMF, Primary myelofibrosis; PV, Polycythemia vera; STAT1, Signal transducer and activator of transcription 1; TLR1, Toll-like receptor 1; TRAF3, Tumor-Necrosis-Factor-receptor-associated factor 3.

## Data Availability

The original contributions presented in this study are included in the article. Further inquiries can be directed to the corresponding author.
